# Supplementary figures and images for: Seroprevalence of SARS-CoV-2 IgG specific antibodies among healthcare workers in the Northern Metropolitan Area of Barcelona, Spain, after the first pandemic wave
Source: PLoS One. 2020 Dec 28;15(12):e0244348. doi: 10.1371/journal.pone.0244348 (PMC7769254; doi:10.1371/journal.pone.0244348)

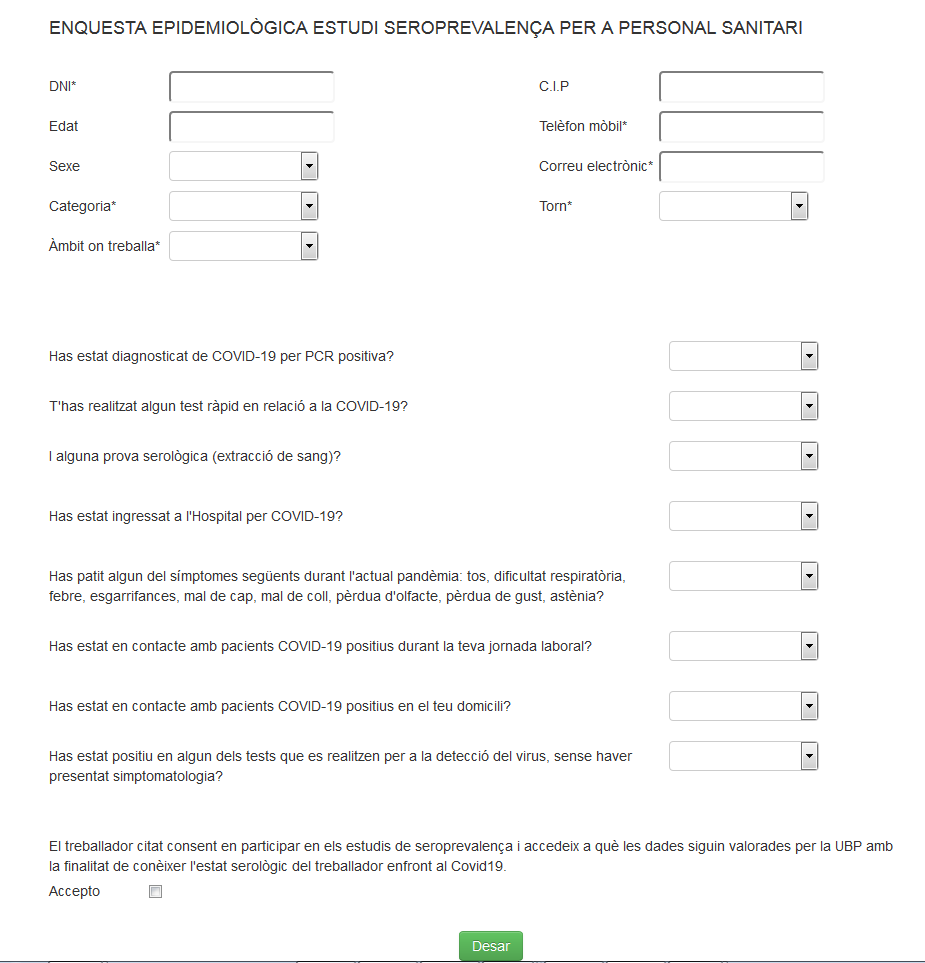

Supplement: S1 File — (TIF) [file pone.0244348.s001.tif]
