# Supplementary material for: Seroprevalence of SARS-CoV-2 IgG specific antibodies among healthcare workers in the Northern Metropolitan Area of Barcelona, Spain, after the first pandemic wave
Source: PLoS One. 2020 Dec 28;15(12):e0244348. doi: 10.1371/journal.pone.0244348 (PMC7769254; doi:10.1371/journal.pone.0244348)
Supplement: S2 File — (PDF) [file pone.0244348.s002.pdf]

EPIDEMIOLOGIC SURVEY
SEROPREVALENCE STUDY FOR HEALTHCARE WORKERS

National identity document:*
Age:
Gender:
Healthcare Job*
Location*

Personal Identification Number
Phone Number*
Email*
Shift*

Have you been diagnosed of COVID-19 with a PCR positive test?

Have you had a rapid test related to COVID-19?

And any serological test (blood extraction)?

Have you required hospitalization because of COVID-19?

Have you experienced any of the following symptoms during the present pandemic: cough, short breath, fever, chills, headache, sore throat, loss of smell, loss of taste, asthenia?

Have you been in contact with COVID-19 positive patients during your work hours?

Have you been in contact with COVID-19 positive patients at home?

Have you been positive for any kind COVID-19 test, without experiencing any symptoms?

The worker consents to participate in the seroprevalence study and accepts that the data will be evaluated by the Disease Prevention Unit (UBP) with the intention of knowing the worker’s COVID-19 serological status.

I accept: ☐
